# A case series of post COVID-19 mucormycosis—a neurological prospective

**DOI:** 10.1186/s41983-021-00355-8

**Published:** 2021-07-26

**Authors:** Tamer Roushdy, Eman Hamid

**Affiliations:** Neurology Department, Faculty of Medicine, Ain Shams University, 38 Abbasia, PO 11591, Cairo, Egypt

**Keywords:** Coronavirus, COVID-19, Mucormycosis, Orbital cellulitis, Orbital apex syndrome, Cavernous sinus thrombosis

## Abstract

**Background:**

Direct neurological manifestations of coronavirus disease whether peripheral or central are reported worldwide. Yet, along the 3rd wave of the pandemic especially in India, an associated angioinvasive opportunistic infection with mucormycosis in COVID-19 cases is emerging.

**Case presentation:**

The current case series which represents 4 patients with mucormycosis post COVID-19 is one of a few if not the first case series that discusses post COVID-19 mucormycosis from a neurological prospective in a tertiary hospital in Egypt.

All cases but one presented with total ophthalmoplegia, and only one was diagnosed as a cavernous sinus thrombosis; meanwhile, orbital cellulitis and orbital apex syndrome were responsible of ophthalmoplegia in two cases.

Mortality reached 25%, and the case that died suffered cutaneous as well as rhino-cerebral type with a delayed presentation to hospital.

**Conclusion:**

A rare but fatal fungal infection is ought to be nowadays kept in mind in COVID-19 active cases as well as in recovered COVID-19 patients, especially those who have comorbid medical conditions as uncontrolled diabetes and who were treated with large doses of corticosteroids.

## Background

Severe acute respiratory syndrome coronavirus 2 (SARS-CoV-2) is besieging the world for more than a year since its declaration by the World Health Organization as a pandemic in March 2020. Its effect on central nervous system has been reported along many studies and reviews either through affecting vascular system in different ways leading to strokes [1] or through retrograde extension to the brain through the olfactory nerve [2].

Olfactory nerve affection in coronavirus disease of the year 2019 (COVID-19) is well known. Anosmia and hyposmia have been reported by many COVID-19 patients worldwide [3].

Yet, it seems that different cranial nerves are being affected by COVID-19 either directly in the context of the acute virus infection phase like the olfactory nerve or as a result of complications related to coronavirus.

In this case series, different cranial nerves involved in 4 cases suffering mucormycosis as an opportunistic fungal infection post COVID-19 infection are presented with a highlight on different anatomical and pathological explanations for such cranial nerve affection (Table 1).

**Table 1:** Clinical characteristics of the cases

	Gender	Age	Medical history	COVID-19 infection date	Time of presentation	Cranial nerves involved	Mucormycosis type (clinical and radiological basis)	Culture/pathology	Fate
Patient 1	F	59	DM	January 2021	21 days post a negative PCR 14 days since sinusitis	II, III, IV, V2, VI, VII	Rhino-orbito-cerebral and cutaneous	Not obtained (died pre-operation)	Died
Patient 2	M	80	DM, HTN, operated cancer colon, chronic renal impairment	April 2021	30 days post a negative PCR Incidental presentation of palatal blackish ulcer	II	Rhino-orbito-cerebral	Not obtained (discharged against medical will before procedure)	Alive
Patient 3	M	73	DM, HTN, ISHD and cardiac stenting	April 2021	14 days post a negative PCR 10 days since sinusitis	III, IV, VI	Rhino-orbito-cerebral	Positive pathology going with mucormycosis	Alive with a lost eye
Patient 4	M	59	DM, HTN	March 2021	10 days post a negative PCR 6 days since sinusitis	III, IV, VI	Rhino-orbito-cerebral	Positive culture (zygomycetes)	Alive

*F* female; *M* male; *DM* diabetes mellitus; *HTN* hypertension; *ISHD* ischemic heart disease; II, optic nerve; III, oculomotor nerve; IV, trochlear nerve; V2, maxillary division of trigeminal nerve; VI, abducent nerve; VII, facial nerve

A formal written consent was obtained from all cases to publish their medical history, laboratory results, and imaging for radiological as well as clinical lesions.

## Case presentation

### Patient 1

A 59-year-old female, with uncontrolled diabetes mellitus (DM), suffered COVID-19 in January 2021 with a positive polymerase chain reaction (PCR) and was managed with broad spectrum antibiotics for bacterial pneumonia on top of viral pneumonitis and corticosteroids in a dose exceeding 90 mg per day.

The patient presented to the hospital 21 days post COVID-19 with 2 weeks duration of unilateral right facial swelling and deviation of angle of the mouth to the left, complete ophthalmoplegia, no perception of light, and ptosis along the right eye.

**Fig. 1:**
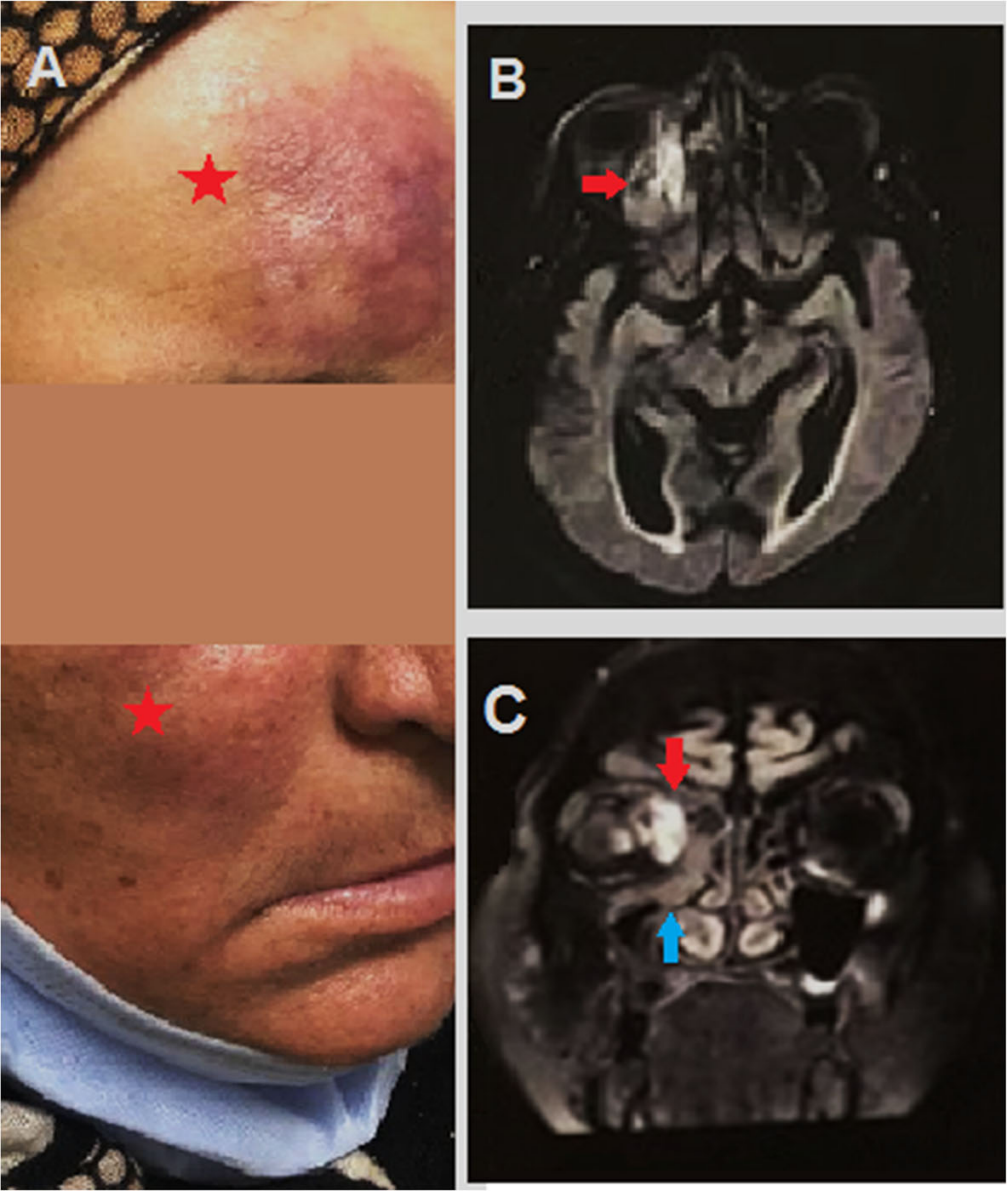
**A** Red star highlighting elevated dark colored forehead and right cheek that goes with cutaneous form of mucormycosis. **B** Red arrow along axial MRI with obliteration of right ostiomeatal complex. **C** Red arrow along coronal MRI revealing obliteration of right ostiomeatal complex with infiltration of infection to the right orbital cavity. Blue arrow showing maxillary, ethmoid and frontal sinusitis all around the right orbital cavity

An elevated skin lesion with dark discoloration along the forehead and right cheek as well as decreased sensation along maxillary division of trigeminal nerve was noted (Fig. 1A).

PCR for COVID-19 was negative; complete blood count (CBC) showed moderate leukocytosis 17.4 × 10^3^/mm^3^ with relative neutrophilia 88% (range 35.0–80.0), absolute neutrophilia 15.31 (range 1.8–7.7) and relative lymphopenia 10.8% (range 18–44), C-reactive protein (CRP) 65 (negative < 6.0), glycated hemoglobin (HbA1C) 12, and serum sodium (Na) 131.4 mmol/L (range 136–145).

Magnetic resonance imaging (MRI) brain and magnetic resonance venography (MRV) with cavernous view as well as MRI orbit with contrast on a 1.5 Tesla super conductive system revealed diffuse enhancement mucosal thickening, along the right maxillary, ethmoidal, and frontal sinuses with occlusion of the right ostiomeatal complex, retro-orbital diffuse edema with faint enhancement, chronic small vessel disease, and intact venous system with no filling defects (Fig. 1B, C).

Direct ophthalmoscope revealed right dilated unreactive pupil, intact anterior segment, and pale disc with suspected retinal artery occlusion.

Dermatology consultation for the forehead lesion provisionally diagnosed it as cutaneous mucormycosis for true cut excision biopsy. Otolaryngology plan was to perform excision biopsy through endoscopic as well as external approach.

The patient was placed on broad spectrum antibiotics, and systemic amphotericin B was initiated. Yet, on the next day prior to the scheduled surgery, the patient suddenly suffered a disturbance in conscious level with a Glasgow coma scale (GCS) 4/15 and was transferred to the intensive care unit (ICU) intubated and ventilated. Later on the same day, the patient died.

### Patient 2

Patient 2 was an 80-year-old male, diabetic, hypertensive, with history of operated cancer colon in 2011 and chronic renal impairment. COVID-19 for this patient was in April 2021 for which he received corticosteroids. The patient presented 30 days later in May 2021 with hard palate sluggish ulcer 1 cm diameter, blackish, and painless not communicating with nasal cavity, with accompanied decrease in visual acuity in the right eye to the degree of seeing objects at 2 m.

PCR for COVID-19 was negative; CBC showed normal leukocyte count 8.2 × 10^3^/mm^3^ with relative neutrophilia 84.1% (range 40.0–80.0) and relative lymphopenia 6.7% (range 20–40), CRP 35 (negative < 6.0), HbA1C 9.5, serum creatinine 2.1, and Na 117 mmol/L (range 136–145).

**Fig. 2:**
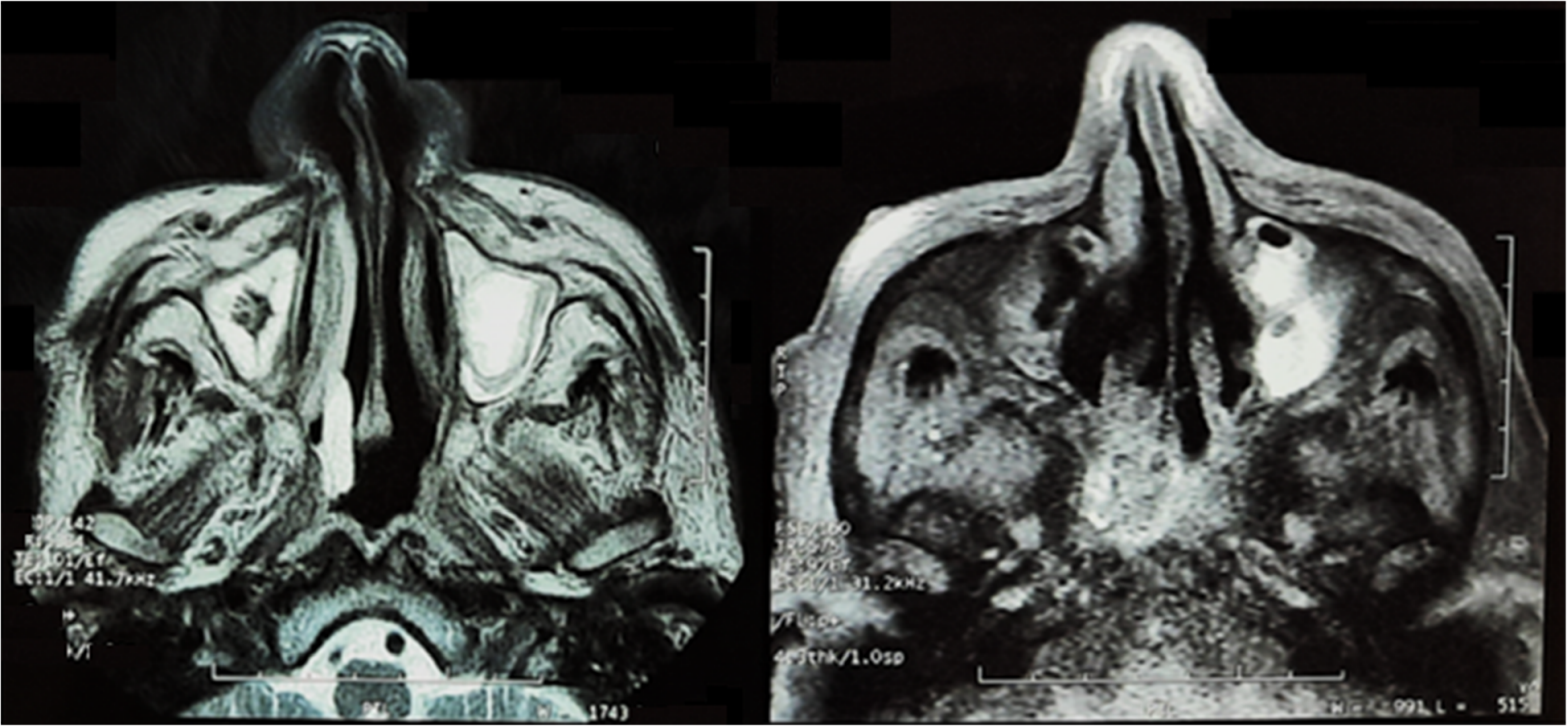
Axial MRI without contrast along the maxillary sinus showing bilateral sinusitis yet more on the left side with hypo intense margins that goes radiologically with fungal infection with heavy metals deposition and hyper intense center that represents bacterial nature

**Fig. 3:**
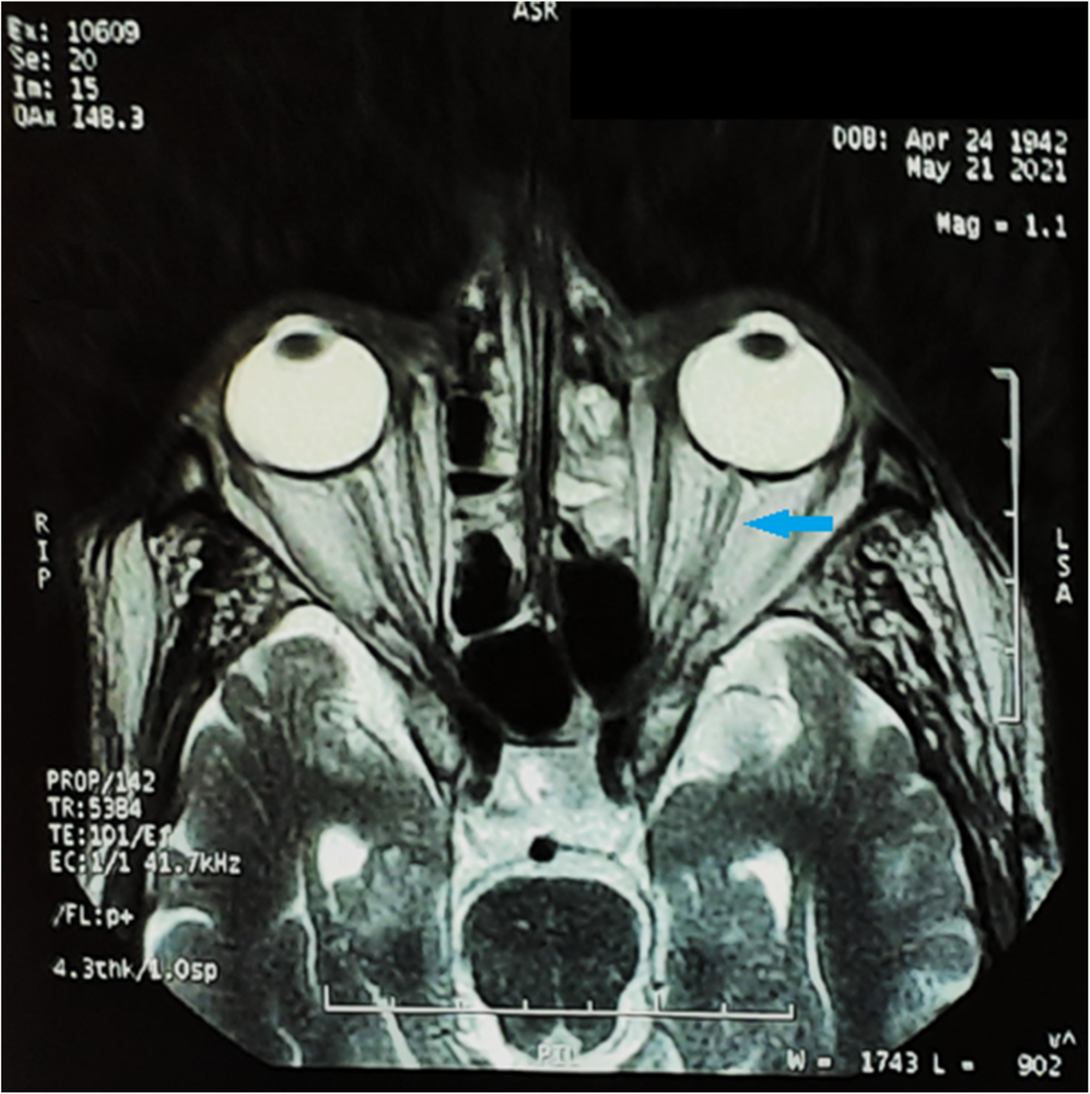
Blue arrow along non contrast MRI T2 WIs highlighting mild form of left optic neuritis

MRI paranasal sinuses performed prior admission at 3 Tesla high field MRI unit revealed near pan sinusitis especially along bilateral frontal, ethmoidal, maxillary sinuses, and left sphenoid. Both ethmoid sinuses show predominantly fungal sinusitis with bacterial sinusitis, while the left maxillary show more bacterial but with fungal component. No abnormal extension of the inflammatory process towards the cavernous sinus regions was noted. Both orbits had normal appearance except for mild form of the left optic neuritis that needed post intravenous contrast study that was inapplicable secondary to renal impairment (Figs. 2 and 3).

The patient was planned for evacuation and drainage of the sinuses under general anesthesia after correcting his hyponatremia.

Broad spectrum antibiotics and systemic amphotericin B were administered. Steroid therapy for optic neuritis was considered yet not initiated upon relative request, and the patient was discharged against medical will.

### Patient 3

A 73-year-old male patient, diabetic, hypertensive, with ischemic heart with coronary stenting, had COVID-19 infection in April 2021 for which he received dexamethasone 8 mg/2 ml ampoule for 7 days and third-generation cephalosporin.

**Fig. 4:**
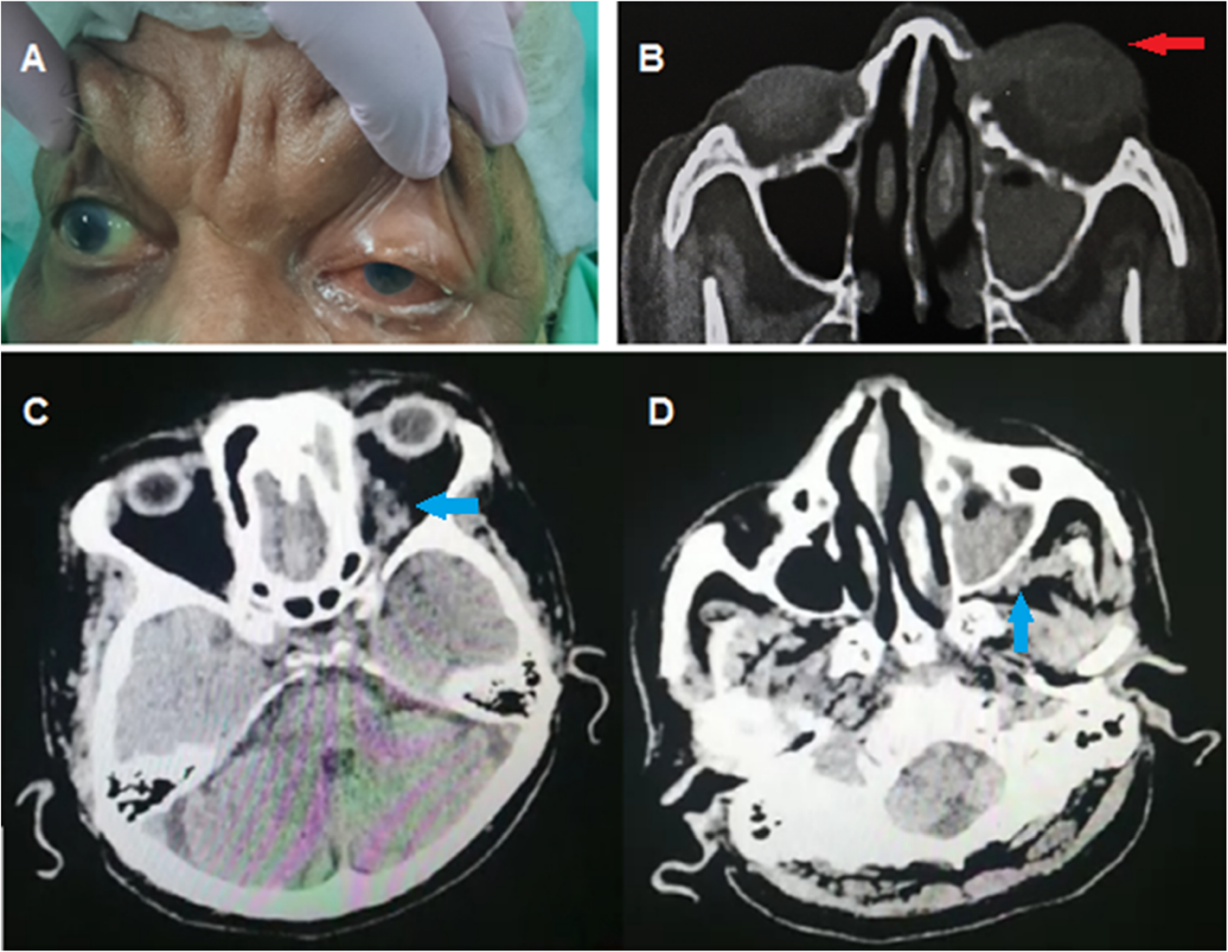
**A** Chemosis and total ophthalmoplegia of the left eye. **B** Red arrow showing proptosis of the left eye along paranasal CT. **C** Blue arrow pointing to infiltration of soft tissue retro orbit sharing in the presentation of orbital apex syndrome donating invasion from infected and inflamed sinuses. **D** Blue arrow representing infiltration of periantral fat donating invasion from nearby sinuses

Two weeks post COVID-19, the patient presented with headache, nasal congestion, complete left ophthalmoplegia, complete ptosis, and conjunctival chemosis (Fig. 4A).

PCR for COVID-19 was negative; CBC was normal, CRP 37.6 (negative < 6), HbA1C 9.5, and Na 127 mmol/L (136–145).

Computed tomography (CT) of the paranasal sinuses without intravenous contrast revealed pan sinusitis much more evident along the left side with obliterated ostiomeatal complex, abnormal fatty stranding surrounding the distal left side optic nerve close to the optic foramen suspicious of orbital cellulitis (Fig. 4).

MRI orbit with contrast revealed diffuse enhancing mucosal thickening with internal fluid level and true diffusion restriction denoting acute sinusitis in the left frontal, ethmoidal, and maxillary sinuses, with left orbital fat diffuse edema with faint enhancement confirming orbital cellulitis.

The patient was placed on systemic and topical eye antibiotics and systemic liposomal amphotericin B in a dose of 0.7 mg/kg intravenous once per day over 6 h for 14 days; drainage and biopsy for the sinuses was performed.

Postoperatively, the patient’s headache improved, yet his eye motion was not regained.

Specimen for an excised infected polypoid lesion was sent for pathological assessment. Microscopic description was in favor of mucormycosis in which there was a partially ulcerated and partially hyperplastic respiratory epithelium. The subepithelial tissue was edematous, congested with dense mixed inflammatory cellular infiltrate rich in neutrophils. Foci of suppuration were seen, with few fungal hyphae, thick walled, non-septate and with irregular branching and occasionally at right angles.

### Patient 4

Patient 4 was a 59-year-old male, with uncontrolled DM, and hypertensive, with history of COVID-19 in March 2021.

**Fig. 5:**
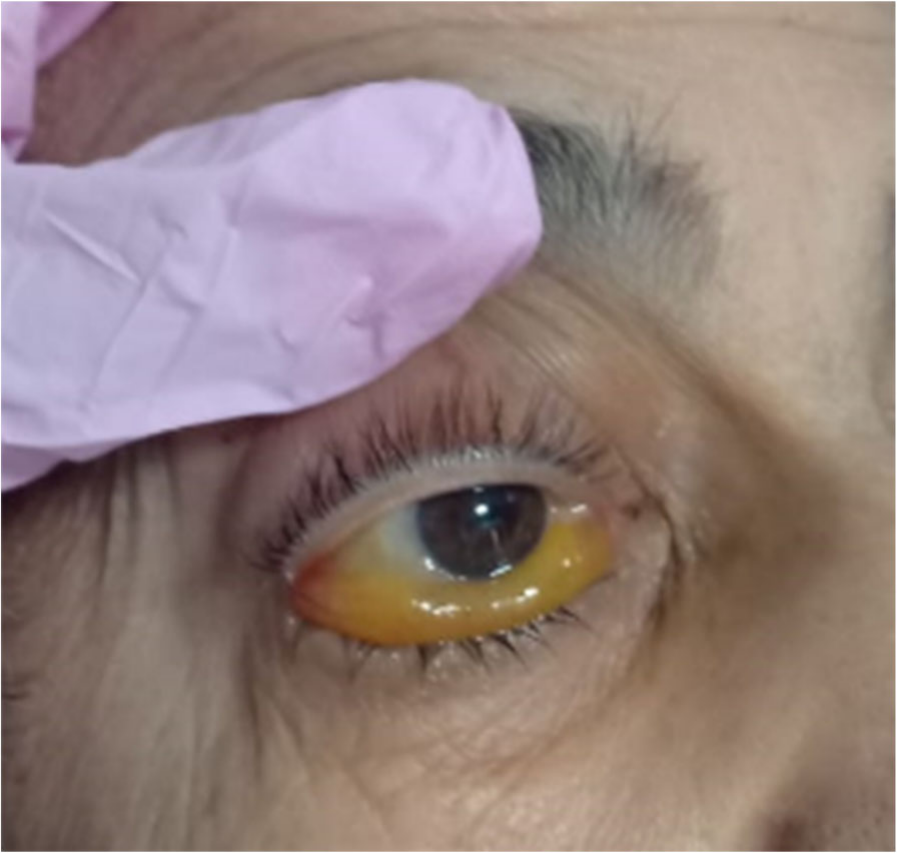
Chemosis of conjunctiva and total ophthalmoplegia with ptosis elevated by the examiner’s finger

The patient presented with 6 days duration of right total ophthalmoplegia, ptosis, conjunctival chemosis, and proptosis (Fig. 5) and blackish discoloration of hard palate.

PCR for COVID-19 was negative; CBC showed moderate leukocytosis 15.3 × 10^3^/mm^3^ with relative neutrophilia 87.9% (range 40.0–80.0), absolute neutrophilia 13.4 (range 2.0–7.0), and relative lymphopenia 1.2% (range 1.0–3.0), erythrocyte sedimentation rate (ESR) 75 mm/h (range 2–20), HbA1C 12.2, and Na 130 mmol/L (136–145).

CT paranasal sinuses revealed pan sinusitis along the right maxillary, ethmoidal, and frontal sinuses with obliteration of the ostiomeatal complex.

MRV cavernous view with contrast showed enhancement and filling defect.

A provisional diagnosis of rhino-cerebral-mucormycosis and associated cavernous sinus thrombosis was considered, and the patient underwent complete drainage of the sinuses; the culture revealed growth of zygomycetes fungi as well as Klebsiella multiple-drug resistant (MDR).

The patient initiated full dose anticoagulant, systemic amphotericin B in a dose of 0.7 mg/kg intravenous once per day over 6 h for a total of 14 days and amikacin antibiotic based on maxillary sinus culture and sensitivity with an excellent recovery and no residual eye motion defect.

## Discussion

COVID-19 infection does not stand only at the acute phase. It has consequences in patients who are either immunocompromised, with uncontrolled diabetes, and who are treated for long periods with high doses of steroids [4].

Opportunistic infection with fungi in nasal sinuses is rare, yet it is being reported nowadays in the COVID-19 pandemic [5]. Mucormycosis caused by mucormycetes molds has five forms based on site of spread being sinuses, orbital and brain (rhino-orbito-cerebral), pulmonary, gastrointestinal, cutaneous, and disseminated [4].

Mucormycosis is diagnosed through different approaches. According to centers for disease control and prevention (CDC), facial swelling, headache, nasal or sinus congestion, and blackish discoloration within nose or palate are a clinical diagnostic approach. Radiological diagnosis is reachable through CT or MRI, and laboratory diagnosis is obtainable either by culture or pathology [6–8].

Along this case series, we have collected 4 cases of post COVID-19 mucormycosis that were presented for suspicion of cavernous sinus thrombosis to neurology department of Ain Shams University Specialized hospital along the first 5 months of 2021 along the third wave of the pandemic.

Four cases in a 5-month duration encountered in a single hospital are considered a relative breakthrough in incidence of such type of rare opportunistic disease. In a retrospective study conducted in children’s cancer hospital 57357 in Egypt along a decade from 2007 to 2017, only 45 cases developed mucormycosis [9]. Another study in 2010 by Zaki and Colleagues that was conducted at the same tertiary hospital where the current case series took place reported 10 cases within 12 months' duration [10].

In the 2010 study, only 2 (20%) cases had a sinus mucormycosis, while in the current case series, 100% of the cases had a sinus infection besides one who had also a cutaneous forehead and cheek type on clinical suspicion.

Laboratory wise, besides the uncontrolled diabetes in the four patients which is considered a risk factor for compromised immunity, the four patients had hyponatremia as a baseline presentation. Low serum Na is a common finding in viral and bacterial infections and is reported in COVID-19 cases. Yet, for fungal infection, it is rarely described [11, 12].

Mucormycosis in the current study is represented from neurological prospective rather than pathological or surgical ones.

Three cases had total ophthalmoplegia, and only one of them was proven to have cavernous sinus thrombosis as a responsible cause for ophthalmoplegia, whereas the other 2 cases total ophthalmoplegia was secondary to direct spread or indirect inflammatory process involving the orbital cavity and causing orbital cellulitis.

Two cases had vision affection: one had a demyelinating signal along the optic nerve shown in non-contrast T2 WIs with a recommendation of post contrast study that was inapplicable secondary to renal impairment. The second patient had dilated unreactive pupil with a suspected retinal artery occlusion in direct ophthalmoscope.

Reduction in visual acuity in cases with mucormycosis is explainable on the basis of infarctions in blood vessels supplying retina or optic nerve, compression on the nerve along its course within cavernous sinus, or direct infection and necrosis [13, 14].

Isolated optic neuritis or retro-bulbar optic neuropathy is infrequently reported with mucormycosis. In the current case series, patient 2 was a probable mucormycosis case based on the European Organization for Research and Treatment of Cancer and Mycoses Study Group (EORTC/MSC) with clinical and radiological signs going with optic neuritis that was a result of inflammatory response rather than direct invasion rather than the one reported by Kahloun and colleagues [15].

Prognosis in mucormycosis is poor secondary to the nature of such opportunistic angioinvasive fungal infection that affects immunocompromised patients with uncontrolled diabetes which is a common risk factor in most cases, besides the delay in presentation to emergency units.

Patient 1 in the current series presented to hospital following 2 weeks duration with cutaneous as well as sinus suspicion of post COVID-19 fungal infection and II, III, IV, V2, and VI cranial nerve affection representing what is termed orbital apex syndrome, besides lower motor neuron facial on top of the swollen face and forehead. The next day and prior to the scheduled operation, the patient suddenly deteriorated and died.

Delayed presentation and combined cutaneous as well as rhino-orbito-cerebral types may rationalize aggressiveness of infection and fatality in such case.

This highlights the importance of time in seeking medical care in such cases, as the other three cases sought medical advice in an earlier course and only one case was left with a lost eye as a lifelong consequence.

A 25% mortality in this collected case series of 5 months is considered high if compared to 10% in 10 cases along Zaki and colleagues’ 12 months duration case series [10].

## Conclusion

Mucormycosis is a rare and occasionally fatal opportunistic infection that affects immunocompromised patients. Most patients who encounter mucormycosis are diabetic with uncontrolled diabetes.

In the COVID-19 era, the rate of mucormycosis seems to be increasing, and the earlier the presentation to hospitals, the better the outcome.

A standard dose recommended by the World Health Organization based on the RECOVERY trial that is 6 mg of dexamethasone once daily for no more than 7–10 days should be strictly adhered to and lower doses should be considered in immunocompromised or diabetic patients.

## Data Availability

The corresponding author takes full responsibility for the data, has full access to all of the data, and has the right to publish any and all data separate and apart from any sponsor.

## Abbreviations

SARS-CoV-2: Severe acute respiratory syndrome coronavirus 2
COVID-19: Coronavirus disease of the year 2019
DM: Diabetes mellitus
PCR: Polymerase chain reaction
CBC: Complete blood count
CRP: C-reactive protein
HbA1C: Glycated hemoglobin
Na: Sodium
MRI: Magnetic resonance imaging
MRV: Magnetic resonance venography
GCS: Glasgow coma scale
ICU: Intensive care unit
CT: Computed tomography
ESR: Erythrocyte sedimentation rate
MDR: Multiple drug resistant
CDC: Centers for Disease Control and Prevention
EORTC/MSC: European organization for research and treatment of cancer and mycoses study group
